# Surgically assisted rapid maxillary expansion in lingual orthodontics – optimizing of coupling and timing: Best oral presentation from the 21st Meeting of German Society of Lingual Orthodontics

**DOI:** 10.1186/s13005-018-0172-6

**Published:** 2018-09-19

**Authors:** Martina Bräutigam, Benedict Wilmes, Nour Eldin Tarraf, Dieter Drescher

**Affiliations:** 1 Department of Orthodontics, University of Duesseldorf, Moorenstr. 5, 40225 Duesseldorf, Germany; 2 Department of Orthodontics, University of Sydney, Private Practice, Sydney, Australia

**Keywords:** Lingual orthodontics, Lingual brackets, SARME, Hybrid-hyrax, Quadhyrax, DW lingual systems

## Abstract

**Background:**

Surgically assisted rapid maxillary expansion (SARME) is primarily used in adult orthodontics. In many cases it is followed by further surgery to address further anteroposterior and/or vertical discrepancies. Treatment times in such cases are often long with adult patients usually requesting invisible appliances. Lingual appliances can provide the mechanical control required as well as fulfil the aesthetic demands in such cases. However lingual appliances are usually custom made and indirectly bonded. Due to tooth movement following surgery there is usually a long delay before impressions can be made for customized lingual appliances. This results in a long delay before alignement and leveling can be commenced post-surgery.

**Case presentations:**

Three cases are presented here demonstrating the simultaneous placement of bone anchored expansion devices for surgically assisted rapid maxillary expansion with customized lingual appliances.

**Conclusions:**

The combination of the two procedures allows the alignement and leveling to commence very soon after surgery significantly reducing treatment times. The design of the appliances and the clinical procedures are described and discussed.

## Background

Surgically assisted rapid maxillary expansion is primarily used to manage transverse maxillary deficiency in adults [1]. Maxillary transverse deficiency usually presents itself with either a bilateral or a unilateral posterior crossbite, the latter often resulting in a mandibular functional shift towards the crossbite side. Ideally this should be addressed with maxillary orthopaedic expansion in growing individuals and if left untreated it can result in a skeletal mandibular asymmetry in adults [2]. Maxillary expansion was described in the dental literature as early as 1860 by Emerson C. Angell in “Dental Cosmos” [3]. Haas later described his method, which is still one of the main methods today [4]. In adults however maxillary expansion can prove difficult with excessive buccal root resorption [5] and gingival recession [6]. This is not only due to the ossification of the midpalatal suture but also due to the resistance form the circummaxillary bones and sutures, which provide the main resistance to expansion [7]. Surgically assisted rapid maxillary expansion SARME was introduced to overcome those difficulties [8]. Surgery usually involves a LeFort I osteotomy with pterygomaxillary disarticulation and midpalatal split.

Three types of expansion appliances have been described with SARME: purely tooth-borne, purely bone borne and tooth-bone borne appliances. With purely tooth-borne appliances there was excessive buccal tipping of the molars [9] as well as root resorption and buccal fenestrations. Bone-borne expansion with the Transpalatal Distractor [10] and the Dresden Distractor by Harzer [11] aimed to minimize the dental side effects and maximize the skeletal expansion. However there remain problems with palatal mucosal irritation as well as risk for root damage in the palatal posterior alveolar process. Additionally with the TPD there is also the need for a palatal incision increasing the risk for complications [1].

**Fig. 1:**
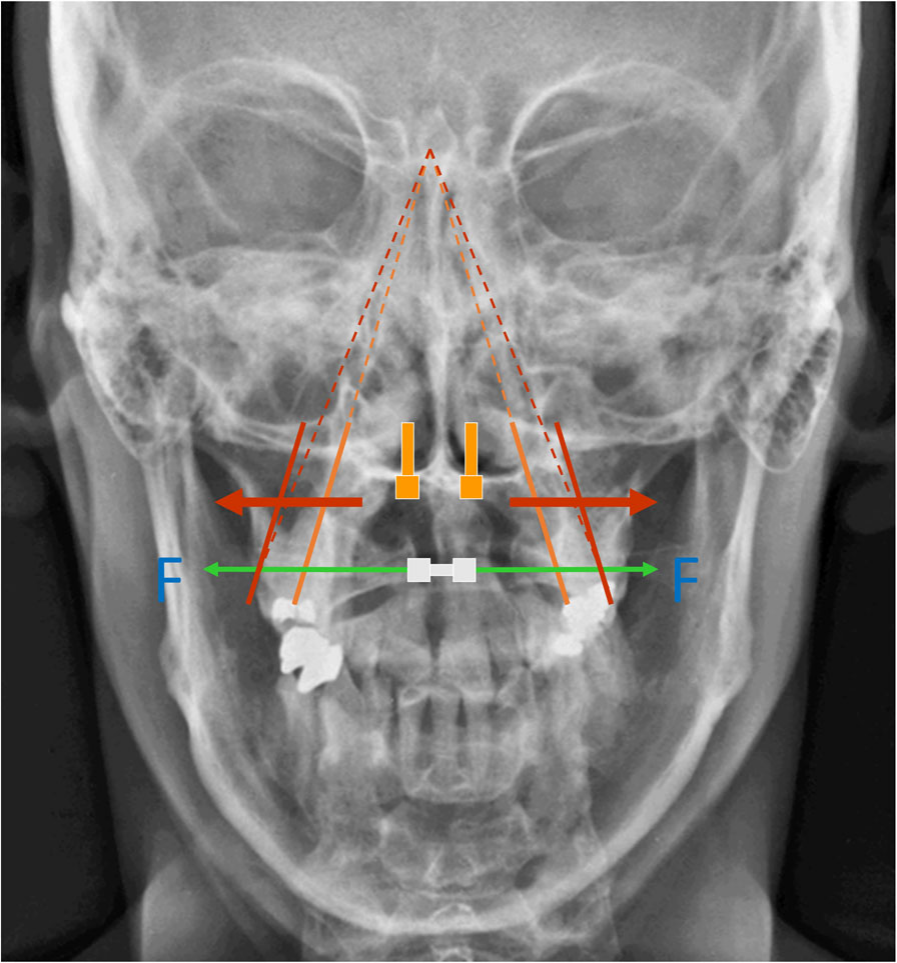
Expansion vector while Rapid Maxillary Expansion. Grey: tooth-borne expansion, orange: bone-borne expansion. The vector is close to the center of resistance with less buccal tipping of the maxillary segments

**Fig. 2:**
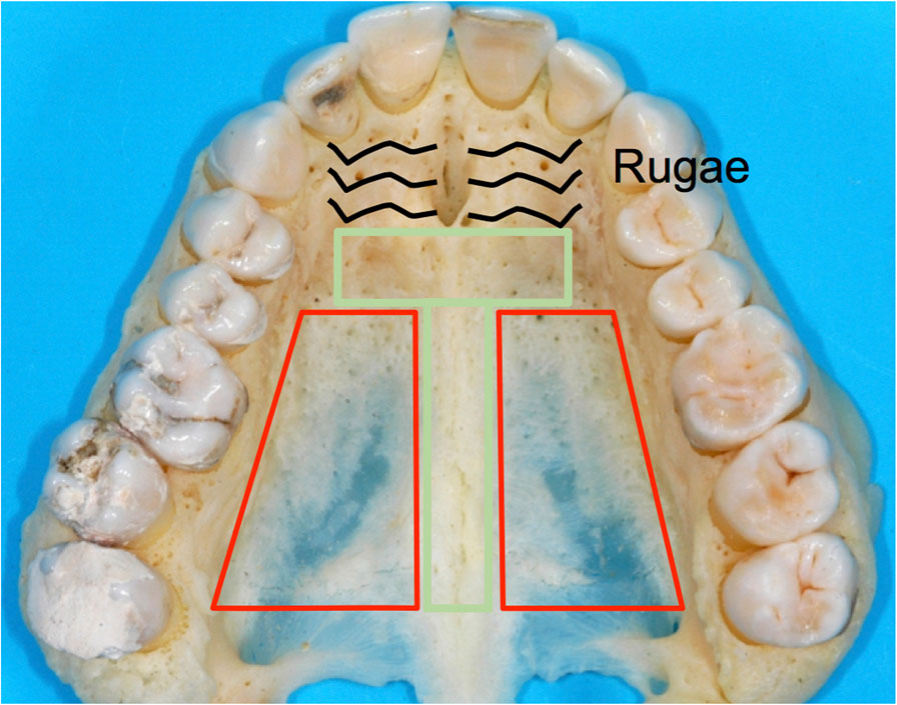
T-Zone: recommended insertion site posterior to the palatal rugae

Wilmes et al. introduced mini-implants with abutments (Benefit system, PSM Medical Solutions, Tuttlingen, Germany) in 2008 [12], which allow mini-implants to be used for skeletal support of expansion. By inserting the mini-implants in the anterior palate, the expansion vector is close to the center of resistance of the maxillary segments [13] (Fig. 1) meaning less buccal tipping of the molars, less resorption of buccal alveolar bone [14], and more basal expansion of the maxilla. The insertion of the palatal mini-implants is minimally invasive with no flap procedures required. For maxillary expansion the insertion is trans-sagittal with the target area for safe placement being the T-Zone immediately posterior to the third palatal Rugae [15] (Fig. 2). In adults predrilling of 2–3 mm is required due to dense cortical bone and 2 mm diameter and 9 mm long mini-implants are used. This placement insures the implants are in the area with the best bone quality while away from the roots of the incisors. The system allows easy coupling with a conventional Hyrax expansion screw through the various abutments available making the laboratory process simple.

SARME is often a first stage surgery to correct transverse maxillary deficiency and further surgery is frequently required after levelling and aligning in order to correct vertical and/or anteroposterior skeletal discrepancies. The extended treatment times in such cases means adults would prefer an invisible appliance. Lingual appliances offer excellent aesthetics as well as the mechanical control required however, because lingual appliances are usually custom made and indirectly bonded, tooth movement following the impressions can lead to ill-fitting appliances and poor results. This is a particular challenge with SARME cases since teeth continue to move for a long duration after expansion especially as anterior teeth drift into the midline diastema. Often there has to be a long delay before impressions can be made for lingual appliances and patients may still have to wear interim retainers to prevent tooth movement until the lingual appliances are ready. This results in unnecessary delay in the alignement and leveling phase following expansion. The following three cases will demonstrate a clinical solution to this problem by simultaneous insertion of the RME device and the lingual appliances prior to surgery. This would allow early levelling of the maxillary arch and thus provide a shorter and more efficient approach while ensuring the accuracy of positioning of the lingual appliances.

## Case presentations

### Case 1

#### Diagnosis and etiology

**Fig. 3:**
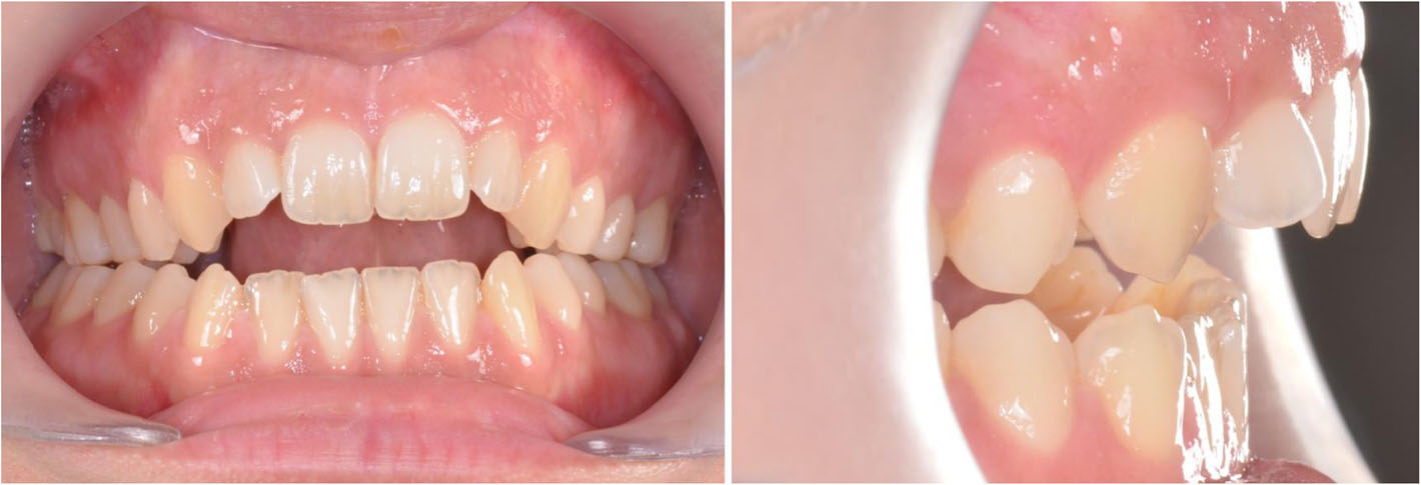
Case 1: Clinical situation before treatment

The first case shows a 30 year old female. Clinical and radiographic examination showed a skeletal Class II pattern with an anterior open bite and a transverse maxillary deficiency with a lateral posterior crossbite on the right and the tendency to a lateral crossbite on the left (Fig. 3).

#### Treatment objectives

The treatment plan involved a first stage of maxillary expansion with SARME to correct the transverse discrepancy followed by the leveling of the dental arches with lingual fixed appliances and finally two jaw surgery to correct the open bite as well as the Class II malocclusion.

#### Treatment procedure

Impressions of the upper and lower arches were obtained for the lingual appliances.

During the planning for the production of the lingual brackets, it was noted that a surgically assisted rapid maxillary expansion takes place. In the set-up, therefore, the transverse width of the upper jaw should be adapted to the lower jaw.

Four Benefit mini-implants were inserted: two in the anterior area of the T-Zone and two 12 mm distally on each side of the midpalatal suture. A silicon impression was taken and the laboratory analogues were placed on the transfer caps. The maxillary expansion appliance was manufactured using a Hyrax screw anchored only to the four mini-implants, named the Quadhyrax.

**Fig. 4:**
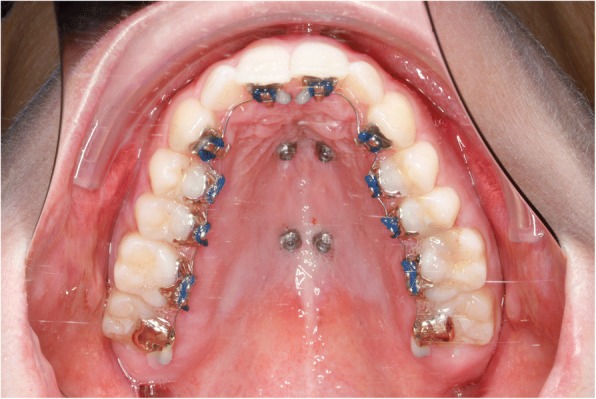
After insertion of four mini-implants and bonding of lingual brackets

**Fig. 5:**
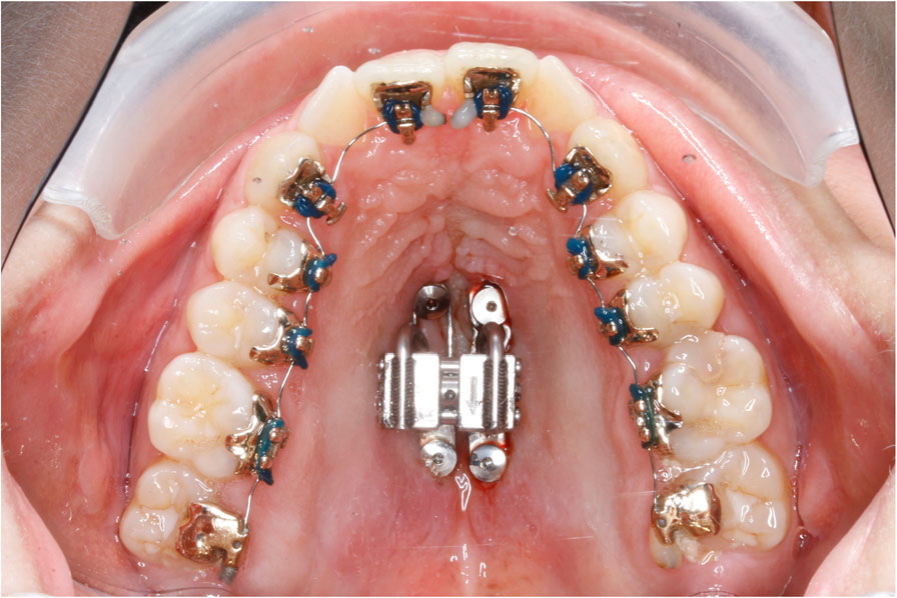
After insertion of Quadhyrax

During the same appointment the lingual appliance was indirectly bonded using a dual cured composite (Fig. 4) and the Quadhyrax was inserted and attached to the mini-implants using Benefit fixation screws. The first lower arch wire 14 NiTi was placed while the upper brackets were securely ligated with a continuous steel ligature in each quadrant to prevent accidental dislodgement during surgery (Fig. 5).

The surgery for SARME was performed on all three patients according to the same procedure: First Le Fort I osteotomy with an oscillating saw. After that, the sutura palatina mediana was chiseled up for the midpalatal split. The tuber region was also mobilized with a chisel for the complete pterygomaxillary disarticulation. The appliance was activated intraoperative to evaluate the individual expansion of both sides of maxilla. After that the aplliance was resetted to reach a final gap of 1 to 1.5 mm.

After surgery and a latency of a few days rapid maxillary expansion commenced with an activation rate of two quarter turns twice a day until the crossbite was corrected [16]. In all three cases one quarter turn corresponded to 0.2 mm. At four turns a day this was equivalent to 0.8 mm.

**Fig. 6:**
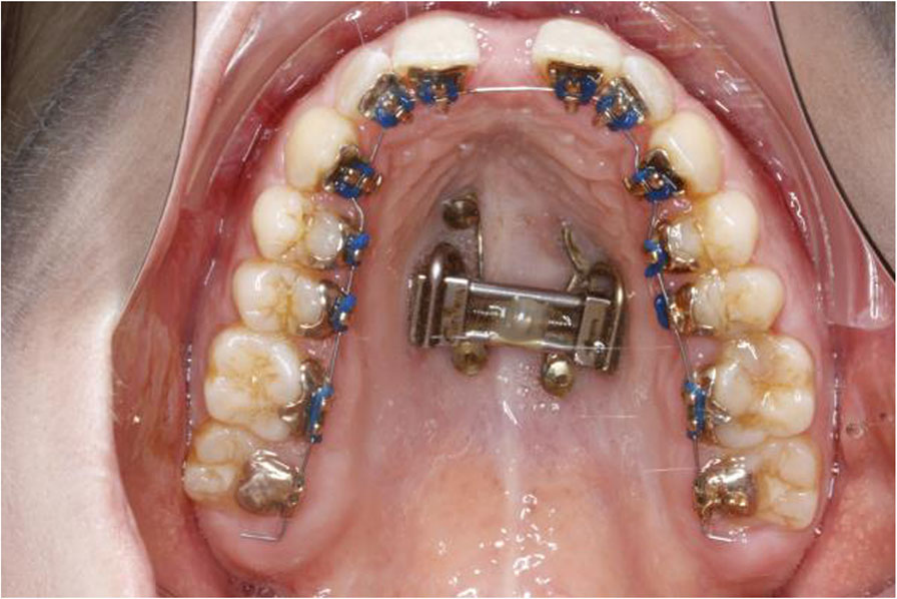
After surgigally assisted rapid maxillary Expansion 14 NiTi archwire is inserted

**Fig. 7:**
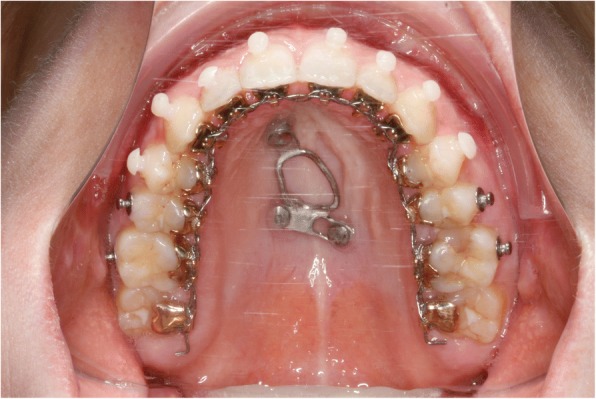
Skeletal retention with 3 mini-implants; situation before orthognathic surgery

**Fig. 8:**
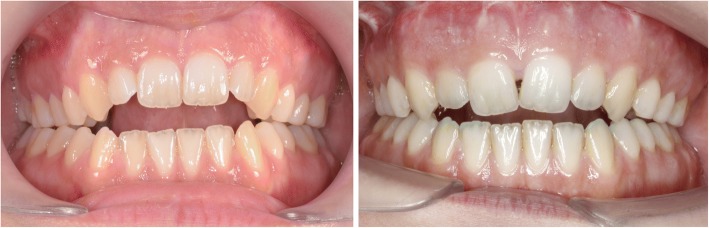
Clinical situation before (left) and after (right) SARME: basal maxillary expansion and palatal tipping of alveolar segments

A central diastema developed and expansion was complete two weeks after surgery. The Hyrax screw was then blocked for retention. Four weeks after surgery the first maxillary archwire 14 NiTi was placed to begin the alignement and leveling phase. The active closure of the central diastema started at about ten weeks post-surgery once enough bone had started to form for the incisors to move into. Because of the typical mushroom shape of the customized lingual appliances, the archwire has to be swiveled using tandem mechanics in front of the canines until the spaces are closed (Fig. 6). The Quadhyrax was removed after six months. One mini-Implant was lost during removal of the expander and the remaining implants served as skeletal retention (Fig. 7). The basal expansion of the maxilla worked well however the tooth-bearing segments of the maxilla showed some palatal tipping (Fig. 8). After successful leveling and radiographic re-examination the second surgery was performed to correct the open bite and the Class II malocclusion.

#### Treatment outcome

The open bite could be closed. The patient has a positive overbite and overjet of 1.5 mm and shows a good transversal and sagittal occlusion.

### Case 2

#### Diagnosis and etiology

**Fig. 9:**
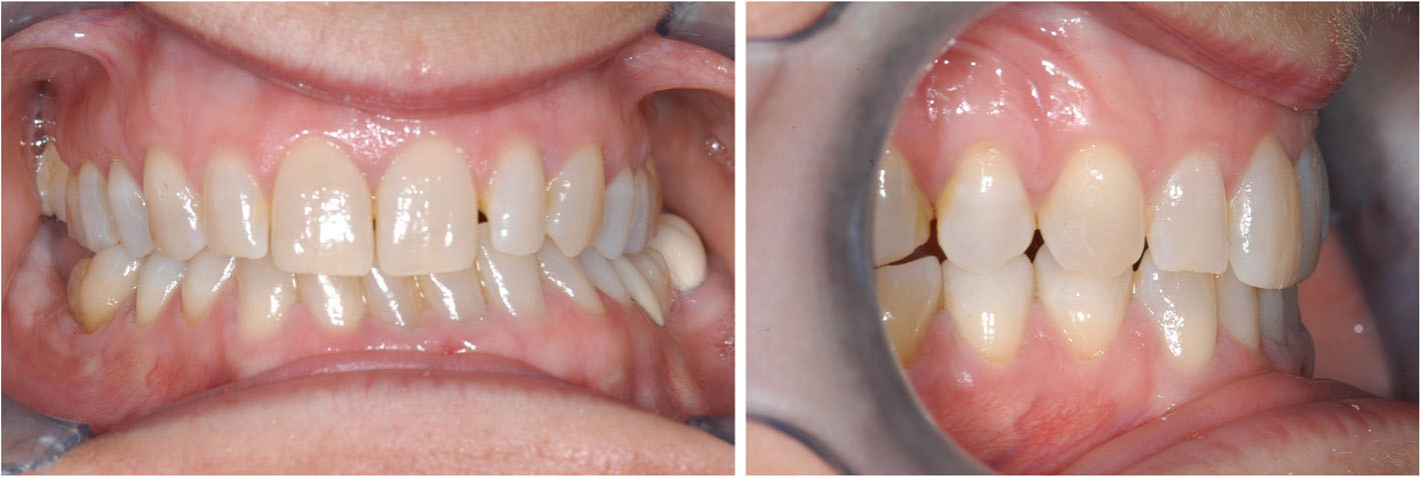
Case 2: Clinical situation before treatment

The second case shows a 53-year-old female. Clinical and radiographic examination confirmed a unilateral posterior crossbite due a transverse maxillary deficiency with a significant mandibular skeletal deviation towards the side of the crossbite (Fig. 9). Treatment objectives.

SARME was planned to correct the transverse discrepancy followed by arch leveling with lingual appliances and then a second surgery to correct the mandibular asymmetry.

#### Treatment procedure

Similar to case 1 impressions were obtained and this time the lingual appliances were manufactured by DW Lingual Systems (Bad Essen, Germany).

During the planning for the production of the lingual brackets, it was noted -similar to case 1- that a surgically assisted rapid maxillary expansion takes place. The transverse width of the upper jaw should be adapted to the lower jaw.

**Fig. 10:**
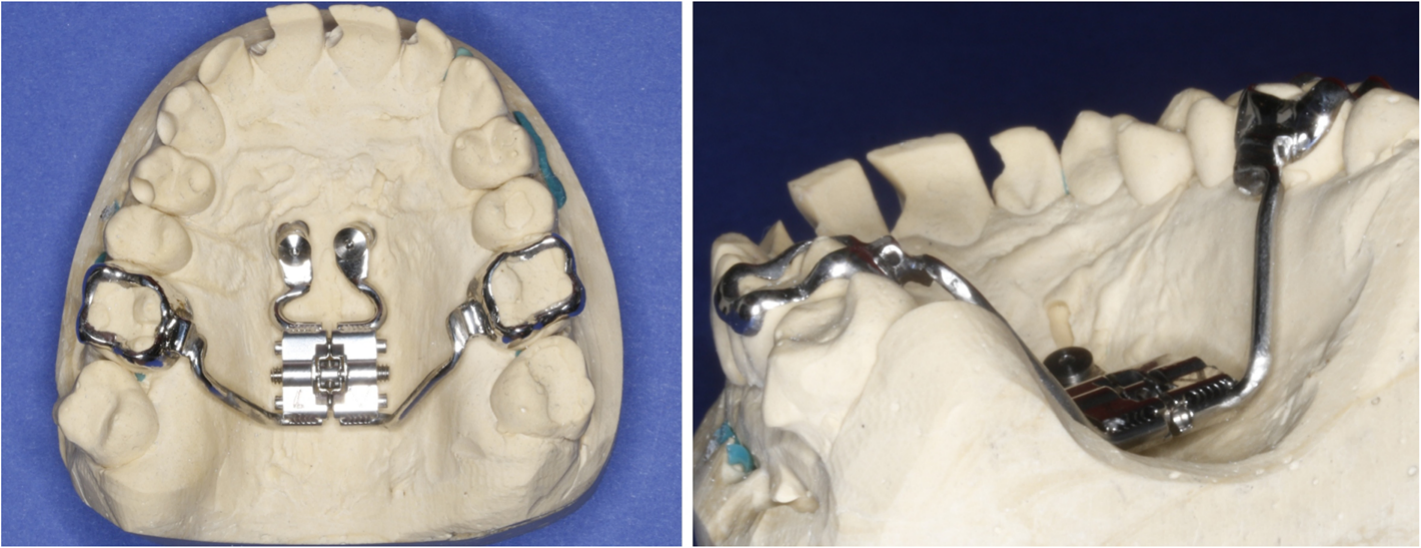
Left: Hybrid Hyrax with lingual bands. Right: Welding coupling of the Hyrax and the molar bands

**Fig. 11:**
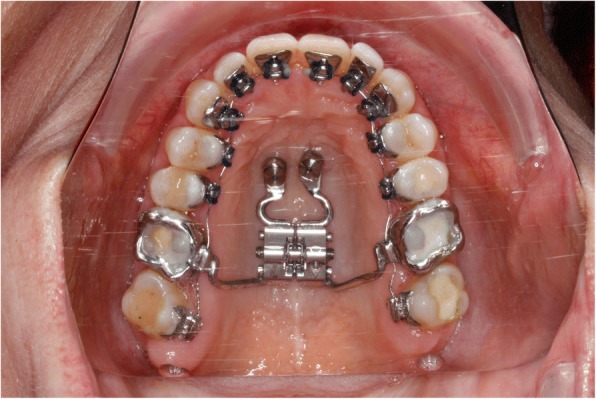
Lingual appliance and Hybrid Hyrax in situ

**Fig. 12:**
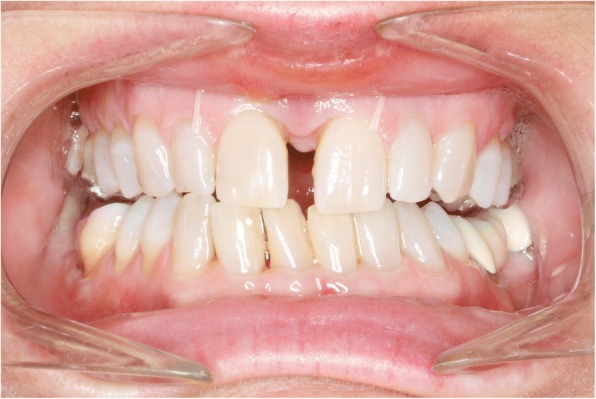
Clinical situation after SARME

**Fig. 13:**
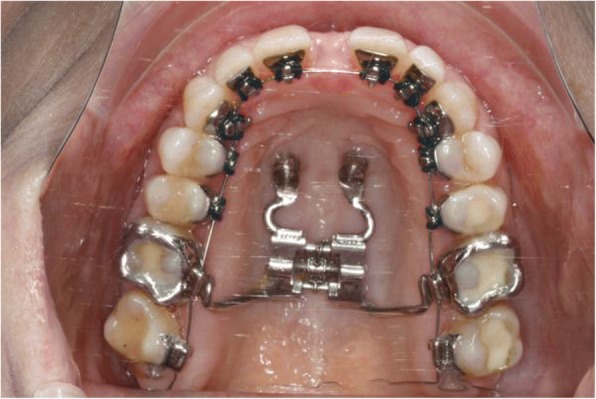
First NiTi archwire after SARME

Two trans sagittal Benefit mini-implants were inserted in the T-Zone. A silicon impression with the transfer caps was taken. The impression was given to the laboratory together with the lingual molar bands. A Hybrid Hyrax [17] was then made and laser welded to the molar bands (Fig. 10). Similar to case 1, the lingual appliance was indirectly bonded with a dual cured resin and the maxillary expansion appliance was inserted. In this case the molar bands were cemented with a dual cured resin and the hybrid hyrax was fixed to the mini-implants using the Benefit fixation screws. The first lower arch wire 12 NiTi was inserted while in the upper the brackets were secured with a continuous steel ligature in each quadrant (Fig. 11). SARME was performed with an activation rate of two quarter turns twice a day until crossbite correction was achieved at two weeks post-surgery (Fig. 12). The Hybrid Hyrax was then blocked. The first upper archwire (12 NiTi) was placed four weeks after surgery (Fig. 13). After complete leveling and radiographic re-examination the surgery to correct the asymmetry was performed.

#### Treatment outcome

The patient has a positive overbite and overjet now. The patient shows a good transversal and sagittal occlusion.

### Case 3

#### Diagnosis and etiology

**Fig. 14:**
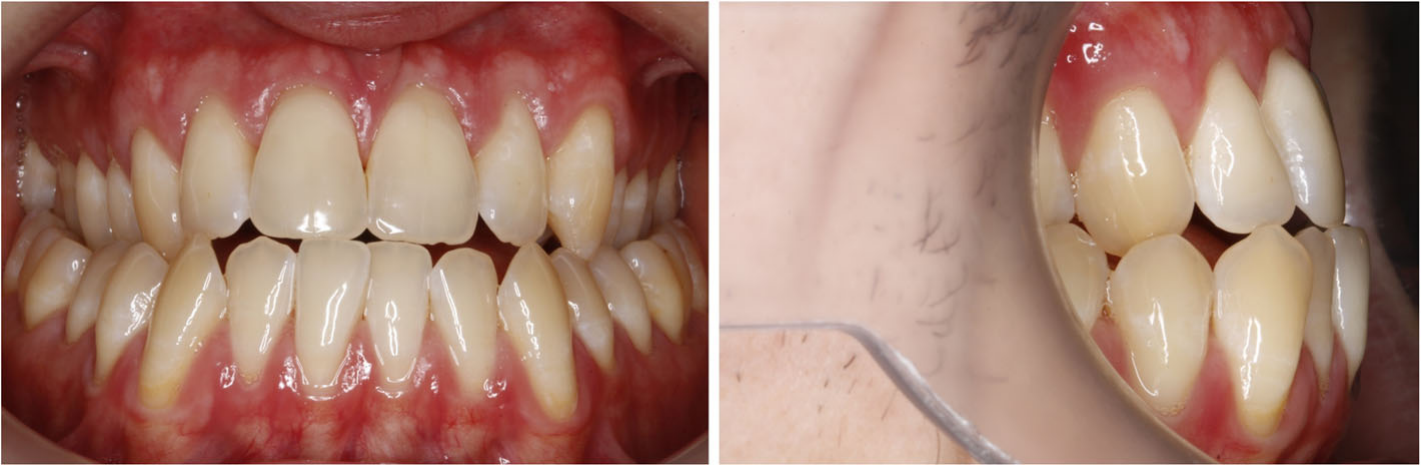
Case 3: Clinical situation before treatment

The third case shows a 30-year-old male. Clinical and radiographic examination confirmed a concave profile, a skeletal Class III pattern with a complete anterior and posterior crossbite. Transverse deficiency of the maxilla was evident with compensatory labial tipping of the upper incisors (Fig. 14).

#### Treatment objectives

Firstly SARME was planned to correct the transverse deficiency. Decompensation was then planned by retraction of the anterior teeth using distalization of the posterior segments and proclination of the lower incisors by leveling. Finally the surgery to correct the Class III malocclusion.

#### Treatment procedure

The insertion-procedure of the mini-implants was similar to case 2. The lingual appliance was also manufactured by DW Lingual Systems (Bad Essen, Germany).

During brackets planning, similar to the previous cases, the transverse width of the upper jaw should be adapted to the lower jaw.

**Fig. 15:**
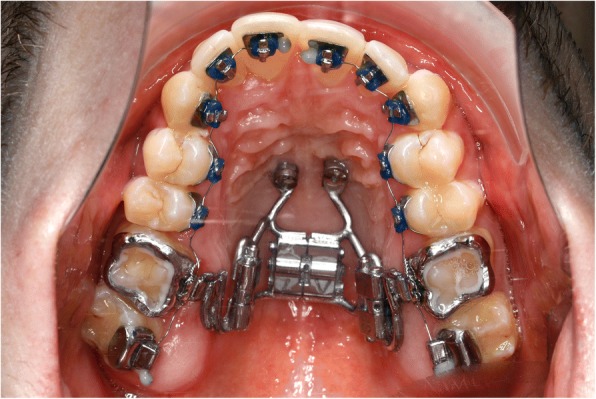
Hybrid-Hyrax-Distalizer with the lingual appliance in situ

**Fig. 16:**
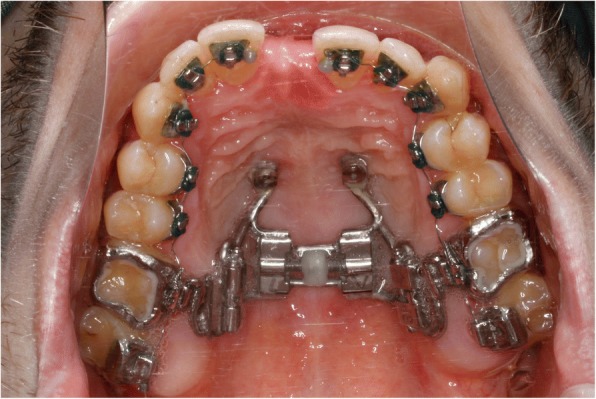
Clinical situation after SARME

**Fig. 17:**
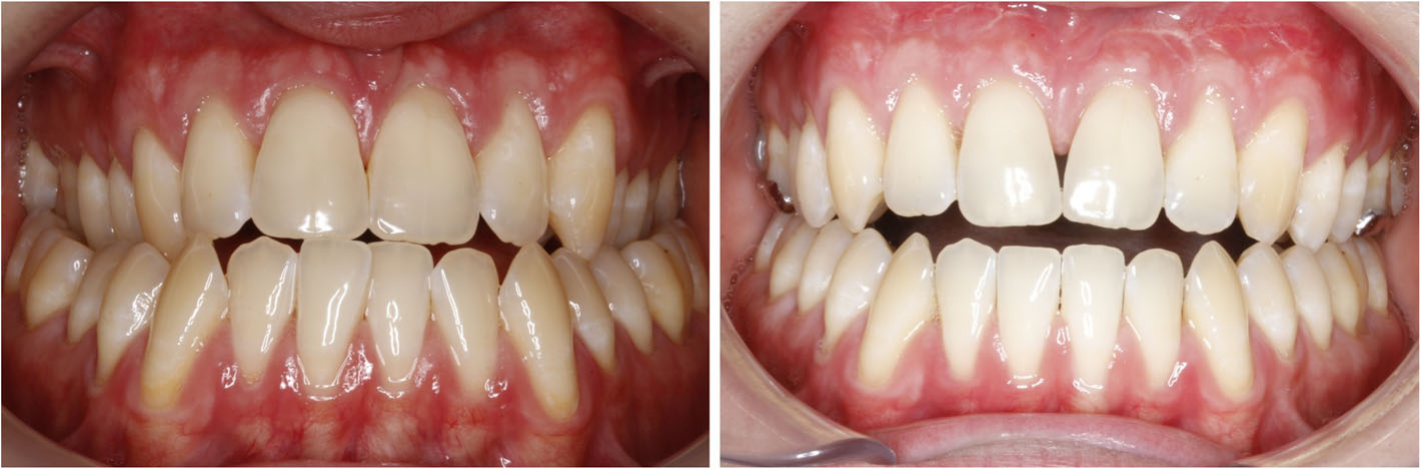
Comparison before (left) and after (right) SARME-treatment

In addition two distalizing-screws were attached between the Hybrid Hyrax and the molar bands (Hybrid Hyrax Distalizer) [18] (Fig. 15). SARME was completed in two weeks with an activation rate of two quarter turns twice a day. The Hybrid Hyrax was then blocked (Fig. 16). Four weeks after surgery leveling was commenced simultaneously with distalization. A 12 NiTi wire was inserted in the upper arch and activation of the distalization screws started at a rate of one quarter turn a week. The active closure of the central diastema started at about ten weeks post-surgery and it was closed one month later (Fig. 17).

**Fig. 18:**
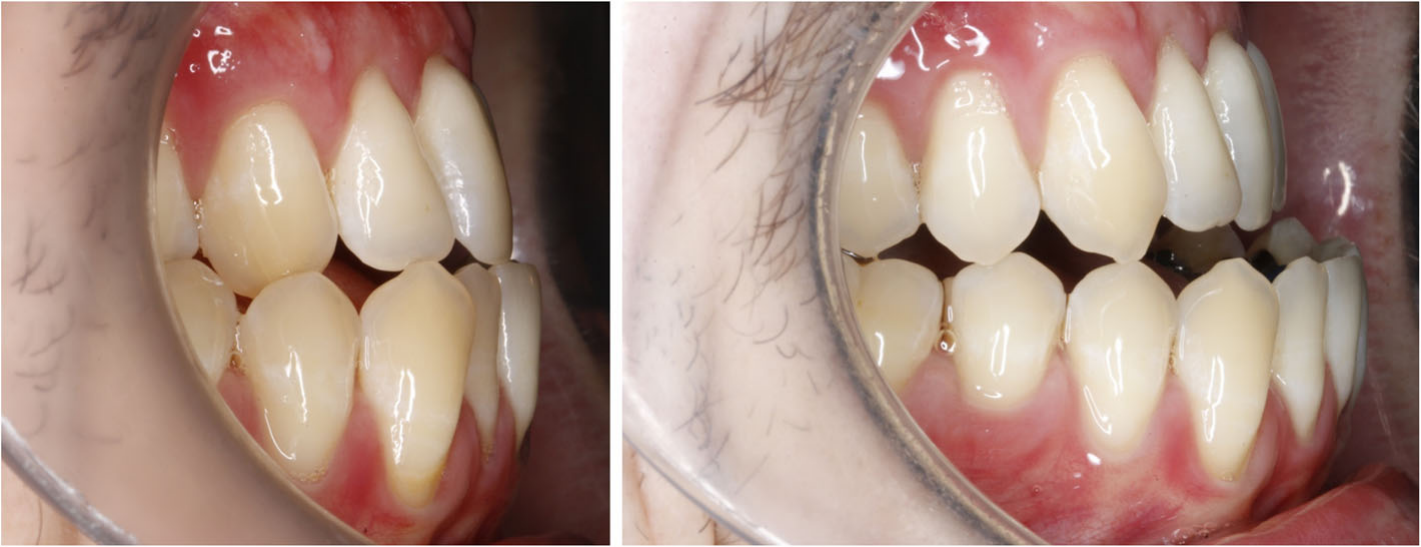
Decompensation: Comparison before (left) and after (right) distalizing

A half year post-surgery radiographic re-examination was made and there was a sufficient distance of repositioning for the jaws (Figs. 18 + 19 + 20) (Tab. 1). The surgery to correct the Class III malocclusion could be performed.

**Fig. 19:**
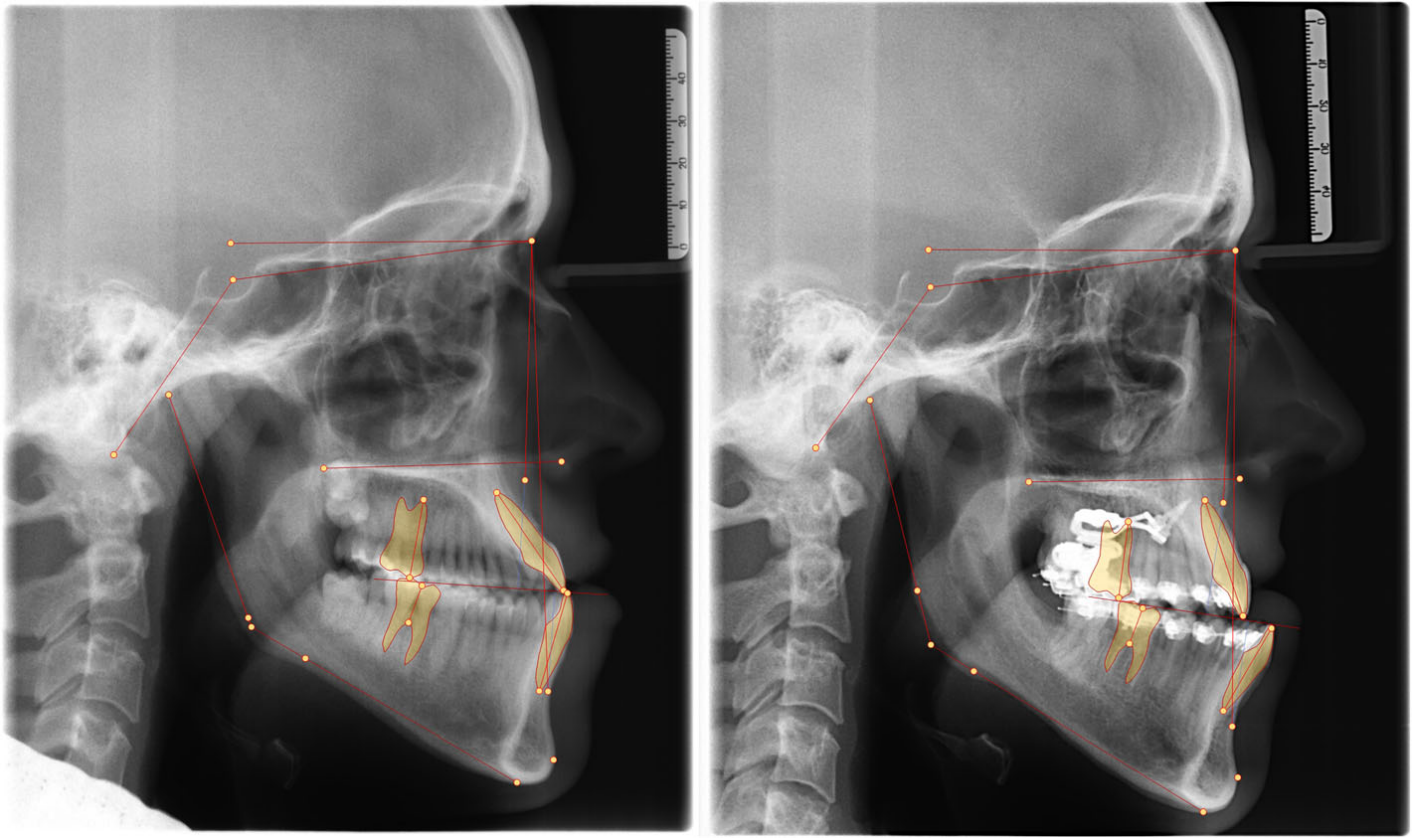
Radiograph comparison before orthognathic surgery: lateral cephalogram pre- (left) and post-distalization (right)

**Fig. 20:**
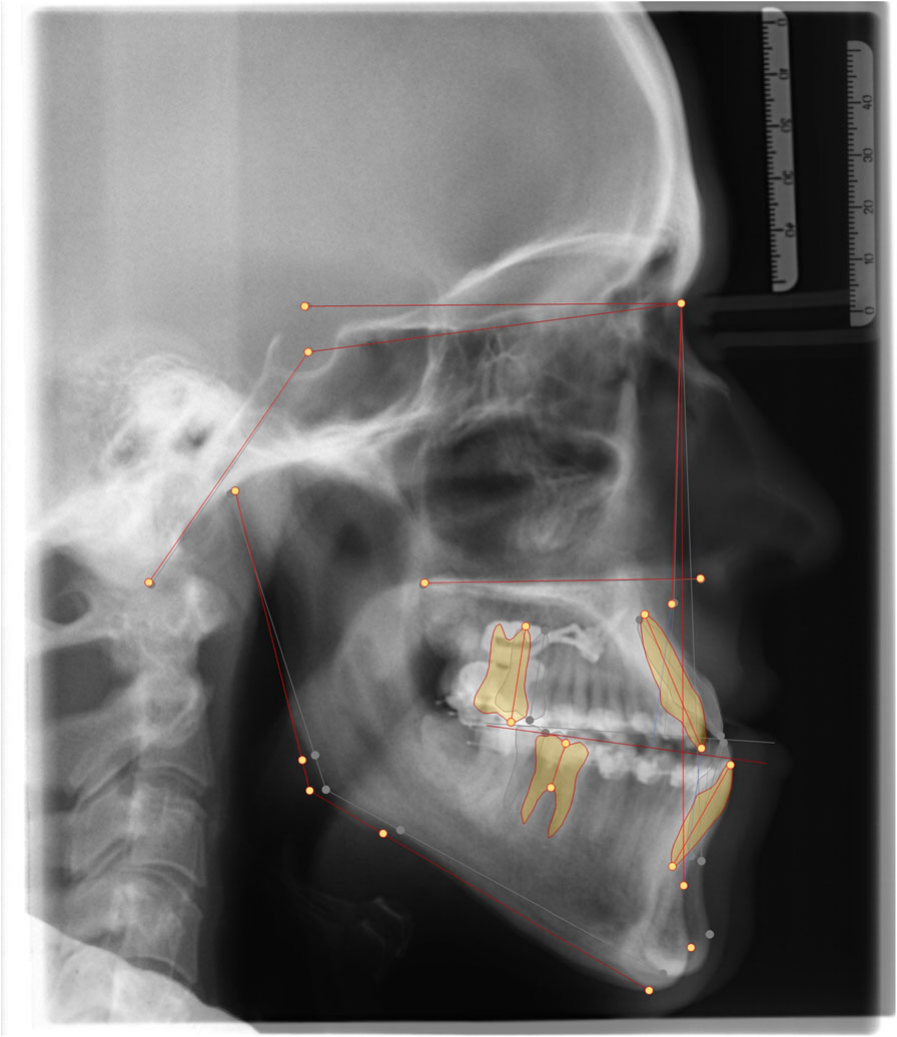
Superimposition of the lateral cephalograms pre- and postdistalization

**Table 1:** Difference between pre- and post-distalisation lateral cephalometric parameters measured

Lateral cephalometric parameters	Pre-distalisation	Post-distalisation	Change
SNA angle (°)	80.9	80.4	−0.5
SNB angle (°)	84.6	82.8	−1.8
ANB angle (°)	−3.7	−2.3	1.4
Wits (mm)	−9.3	−9.8	−0.5
ML-NL (°)	31.9	32.3	0.4
UI-NL (°)	123.1	110.1	−13.0
LI-ML (°)	75.7	88.8	13.1

Key: *SNA* angle between Sella-Nasion-A point; *SNB* angle between Sella-Nasion-B point;

*ANB* difference SNA-SNB; Wits: measure of sagittal jaw discrepancy at occlusal leve;

*NL* palatal plane; *ML* mandibular plane; *UI* upper incisor long axis; *L1* lower incisor long axis

#### Treatment outcome

The patient has a positive overbite and overjet now. The patient shows a good transversal and sagittal occlusion.

## Discussion

Surgically assisted maxillary expansion was introduced to manage transverse maxillary skeletal deficiency in adults [8]. In many cases SARME is a first stage surgery followed by alignement and leveling then a second stage surgery is performed to correct further anteroposterior and/or skeletal discrepancies. The treatment times in such cases are often long and most adult patients will demand invisible appliances. Lingual appliances are very well suited for such cases as they offer the necessary aesthetics as well the precise mechanical control needed for such cases. However lingual appliances are usually custom made on an individual laboratory setup. Even though the technique shows a great degree of precision in delivering the desired setup [19], accurate transfer of the bracket position from the laboratory setup to the patients mouth is crucial for the correct expression of the desired setup and the success of the treatment. For this reason even the most minute tooth movement between impression taking and bonding can result in an ill-fitting appliance and thus a poor result. This can be a particular challenge in cases with SARME. Following maxillary expansion the teeth will continuously move for an extended period of time especially as the anterior teeth drift into the created midline diastema. This means that an extended delay is required before impressions for lingual appliances can be made. Additionally patients will have to wear an interim retainer to prevent further tooth movement till the appliances are ready [20]. This also delays and interferes with the spontaneous closure of the central diastema that most patients find aesthetically distressing. The above cases demonstrate a good solution to this problem. By obtaining the impressions and inserting the lingual appliances prior to the surgical expansion accurate bracket positioning of the lingual appliances is guaranteed and thus a precise delivery of the desired setup. There is also a significant shortening in the overall treatment time since alignement and leveling can commence very soon after the surgery without any delays waiting for stabilization, laboratory turnaround or retainers. Additionally active closure of the diastema can also commence once the initial bone healing has taken place [21].

Furthermore the use of a bone-borne expander allowed for the entire dental arch to be accurately bonded with the lingual appliances prior to commencement of treatment unlike with tooth-borne expanders where bonding of the premolars and molars would have to be delayed. In the two cases where a Hybrid Hyrax was used the coupling of the lingual molar bands with Hyrax still made it possible to have the molar brackets present from the beginning of treatment.

Moreover once expansion is completed the molars can be released from the hybrid hyrax and the expander itself can be left in situ as a retainer for an extended period of time until ossification and healing is complete. As an alternative, the expander can be removed and purely skeletal retention can be achieved using the palatal mini-implants and a custom made plate (case 1) or a prefabricated Beneplate [22]. Unlike tooth borne appliances the retention is independent from the teeth and thus early levelling of the dental arch can commence which offers further time savings.

There was a slight difference in the expansion observed with the two expansion designs used above. Bone-borne expansion was originally introduced to overcome some of the problems with buccal tipping of the bony segments [23], which reduces the amount of skeletal expansion and introduces relapse. Root resorption and alveolar fenestration were also a problem with tooth borne expanders. Similar to what has been reported with the TPD [23, 24] and the Dresden Distractor there was more basal bone expansion with palatal tipping of the dental segments with the purely bone-borne Quadhyrax. This means that this design may be better used in cases where there is need for more basal bone expansion and less expansion at the level of the dental arch. The expansion observed with the tooth-bone-borne hybrid hyrax showed more increase in the dental arch perimeter with more bodily expansion of the segments thanks to the rigid connection with the WIN molar bands and the Hybrid Hyrax only a small amount of over correction seemed necessary. This would make this a more efficient design for cases where increase in arch perimeter and dental expansion is of importance. Additionally only two mini-implants are required.

## Conclusions

The above cases demonstrated that simultaneous insertion of the bone-borne RME and the lingual appliances was and effective and efficient treatment protocol. The combination reduces treatment time and allows early alignement and leveling following maxillary expansion while allowing accurate placement of the lingual brackets. The use of bone-borne and tooth-bone-borne expansion allows for effective skeletal expansion with minimal dental side effects.

## Abbreviations

SARME: Surgically assisted rapid maxillary expansion
